# Aktuelle dermatologische Leitlinien in Deutschland und Europa: Eine praxisorientierte Übersicht ausgewählter Empfehlungen

**DOI:** 10.1007/s00105-021-04775-8

**Published:** 2021-03-05

**Authors:** A. Nast, R. N. Werner, C. Dressler, M. Zidane, A. Heratizadeh, M. Gaskins

**Affiliations:** 1 Division of Evidence-Based Medicine (dEBM), Klinik für Dermatologie, Venerologie und Allergologie, Charité – Universitätsmedizin Berlin, Charitéplatz 1, 10117 Berlin, Deutschland; 2 Abteilung für Immundermatologie und experimentelle Allergologie, Klinik für Dermatologie, Allergologie und Venerologie, Medizinische Hochschule Hannover, Hannover, Deutschland

**Keywords:** Dermatologie, Therapie, Diagnostik, Haut, Entscheidungshilfe, Dermatology, Therapy, Diagnosis, Skin, Decision aids

## Abstract

Medizinische Leitlinien sind systematisch entwickelte Entscheidungshilfen für die angemessene Vorgehensweise bei speziellen gesundheitlichen Problemen. Dermatologische Leitlinien in Deutschland werden führend von der Deutschen Dermatologischen Gesellschaft in Kooperation mit dem Berufsverband Deutscher Dermatologen herausgegeben. Darüber hinaus werden auch europäische und internationale dermatologische Leitlinien erstellt, u. a. durch das im Jahr 2018 vom European Dermatology Forum gegründete European Centre for Guidelines Development. In den Jahren 2019 und 2020 wurden unter anderem deutsche Leitlinien zu den Themen pathologische Narben (hypertrophe Narben und Keloide), kutaner Lupus erythematodes, Pyoderma gangraenosum, analer Pruritus, Analekzem, Analkanal- und Analrandkarzinome, Impfprävention HPV(humane Papillomviren)-assoziierter Neoplasien sowie Syphilis und Systemtherapie bei Neurodermitis überarbeitet oder neu erstellt. Auf der europäischen Ebene schließt die Leitlinie zum Lichen planus eine in Deutschland noch bestehende Lücke der vorhandenen Leitlinien. Schlüsselempfehlungen und wesentliche Neuerungen in den Leitlinien werden hier vorgestellt.

## Abstract (en)

Clinical practice guidelines are systematically developed decision aids for specific medical conditions. In Germany, national dermatology guidelines are developed chiefly under the aegis of the German Dermatological Society in collaboration with the Professional Association of German Dermatologists. European and international dermatological guidelines also exist and are developed by a range of organisations, such as the European Centre for Guidelines Development, which was founded by the European Dermatology Forum in 2018. In the years 2019 and 2020, new or updated German national guidelines were published on topics such as pathological scars (hypertrophic scars and keloids), cutaneous lupus erythematosus, pyoderma grangrenosum, anal pruritus, anal eczema, anal canal and anal rim carcinomas, as well as the prevention of HPV-associated neoplasms through vaccination, syphilis and the systemic treatment of neurodermitis. A new European guideline on lichen planus closes a gap in the spectrum of guidelines available in Germany. Key recommendations and relevant changes in the guidelines are presented in this article.

Medizinische Leitlinien sind nach Definition der Bundesärztekammer „systematisch entwickelte Entscheidungshilfen über die angemessene ärztliche Vorgehensweise bei speziellen gesundheitlichen Problemen“ ([1], S. A-2154). Gegenüber Leitlinien sind Richtlinien abzugrenzen. Diese sollten „Regelungen des Handelns oder Unterlassens vorbehalten bleiben, die von einer rechtlich legitimierten Institution konsentiert, schriftlich fixiert und veröffentlicht wurden, für den Rechtsraum dieser Institution verbindlich sind und deren Nichtbeachtung definierte Sanktionen nach sich zieht“ ([1], S. A-2154).

Die Deutsche Dermatologische Gesellschaft (DDG) erstellt und aktualisiert in Zusammenarbeit mit dem Berufsverband Deutscher Dermatologen (BVDD) kontinuierlich die dermatologischen Leitlinien in Deutschland und beteiligt sich an interdisziplinären Leitlinien anderer Fachgesellschaften. Auf der Homepage der Arbeitsgemeinschaft der Wissenschaftlichen Medizinischen Fachgesellschaften e. V. (AWMF) befinden sich aktuell 113 Leitlinien, die unter Führung oder Beteiligung der DDG erstellt wurden (Stand November 2020). Die Leitlinien werden nach den methodischen Vorgaben der AWMF erstellt und entsprechend ihrer Entwicklungsstufe in S1 bis S3 klassifiziert (Tab. 1; [2]).

**Table 1:** 

S3	Konsens- und evidenzbasierte Leitlinie	Repräsentatives Gremium; systematische Recherche, Auswahl und Bewertung der Literatur; strukturierte Konsensfindung
S2e	Evidenzbasierte Leitlinie	Systematische Recherche, Auswahl, Bewertung der Literatur
S2k	Konsensbasierte Leitlinie	Repräsentatives Gremium, strukturierte Konsensfindung
S1	Handlungsempfehlungen von Expertengruppen	Konsensfindung in einem informellen Verfahren

Einige europäische Organisationen, wie z. B. das European Dermatology Forum (EDF) und die International Union against Sexually Transmitted Infections (IUSTI), erstellen auch europäische dermatologische Leitlinien. Vom EDF wurde im Jahr 2018 das European Centre for Guidelines Development in Zusammenarbeit mit der Division of Evidence-Based Medicine an der Charité – Universitätsmedizin Berlin gegründet, um die Entwicklung qualitativ hochwertiger dermatologischer Leitlinien durch eine Harmonisierung und Systematisierung von Definitionen und Methoden in der Leitlinienentwicklung auf europäischer Ebene zu fördern [3].

Dieser Review fasst eine Auswahl von dermatologischen Leitlinien bzw. Leitlinien mit dermatologischer Beteiligung aus den Jahren 2019 und 2020 zusammen.

## Leitlinien unter Federführung der Deutschen Dermatologischen Gesellschaft

### S2k-Leitlinie „Therapie pathologischer Narben (hypertrophe Narben und Keloide)“ [4]

Die Sk2-Leitlinie zur Therapie pathologischer Narben (hypertrophe Narben und Keloide) wurde aktualisiert und hierbei um neue Therapieoptionen erweitert. So finden jetzt der fraktionierte ablative Laser, das Microneedling und die intraläsionale Kryochirurgie Erwähnung. Zudem werden auch Narben nach Verbrennungen und Verbrühungen in der Aktualisierung genauer abgehandelt. Hierbei werden insbesondere die ablativen fraktionierten Laser und das Microneedling empfohlen. Für nicht aktive flache Keloide kann eine Therapie mittels ablativen Lasers in Kombination mit Triamcinolon-Injektion oder als „laser-assisted drug delivery“ erwogen werden.

Die wichtigsten Behandlungsoptionen bleiben die Injektion von Triamcinolon in Kristallsuspension und die Kryotherapie, die in der Regel in Kombination angewendet werden sollen. Operative Maßnahmen werden nur nach intensiver Abwägung und mit zusätzlicher Anschlussbehandlung (z. B. Bestrahlung, Druck, Triamcinolon-Injektion) empfohlen. Die Leitliniengruppe hebt den Empfehlungsgrad für 5 Fluoruracil („off label“) bei therapieresistenten Keloiden von „kann erwogen werden“ auf „kann empfohlen werden“. Die Erfahrungen zu dieser Intervention nehmen zu, und es findet eine vermehrte Anwendung auch in Deutschland statt. Die positiven Studiendaten, die überwiegend aus den englischsprachigen Ländern kommen, haben sich nun auch in der klinischen Anwendung in Deutschland widergespiegelt. Die Leitlinie hat erstmalig auch die Therapieoptionen Hyaluronidase, Kalziumkanalblocker und Plasma aufgenommen. Aktuell kann noch keine Empfehlung für oder gegen die Anwendung dieser Therapien zur Prophylaxe oder Therapie von hypertrophen Narben oder Keloiden aufgrund der geringen sowie oftmals widersprüchlichen Datenlage gegeben werden. Die Erwähnung in der Leitlinie lädt aber zu klinischen Studien ein und stellt den Forschungsbedarf dar.

### S2k-Leitlinie „Diagnostik und Therapie des kutanen Lupus erythematodes“ [5]

Aufbauend auf der deutschen S1-Leitlinie und der europäischen S2k-Leitlinie wurde eine S2k-Leitlinie zu Diagnostik und Therapie des kutanen Lupus erythematodes (CLE) erstellt.

Die Diagnostik des CLE sollte auf Grundlage der klinischen und histologischen Befunde der Patient*innen erfolgen. Um die klinische Diagnose zu bestätigen, wird eine Biopsie idealerweise aus einer aktiven und nicht vortherapierten Läsion zur histologischen Sicherung empfohlen. In differenzialdiagnostisch schwierigen Fällen kann die Durchführung einer direkten Immunfluoreszenz und einer standardisierten Photoprovokation empfohlen werden.

Neben textilem Lichtschutz werden die konsequente Anwendung von Lichtschutzpräparaten an sonnenexponierten Stellen sowie eine absolute Nikotinkarenz empfohlen.

Neben präventiven Maßnahmen wird bei lokal begrenzten Hautmanifestationen zunächst eine topische Therapie (Kortikosteroide, Calcineurininhibitoren) und bei unzureichendem Ansprechen eine Ergänzung um Hydroxychloroquin oder Chloroquin empfohlen. Bei schweren und ausgeprägten Hautmanifestationen wird als Erstlinientherapie eine Kombination aus topischer Therapie, Hydroxychloroquin oder Chloroquin (aktuell in Deutschland außer Handel) und systemischen Kortikosteroiden empfohlen. Als Zweitlinientherapie können bei partiellem Ansprechen Methotrexat (MTX), Retinoide und Dapson (jeweils zu der bestehenden Therapie mit Hydroxychloroquin/Chloroquin) ergänzt werden.

Bei aktiver Erkrankung während der Schwangerschaft oder Stillzeit wird Hydroxychloroquin in der Erstlinientherapie des CLE empfohlen. Eine bestehende Hydroxychloroquin-Therapie sollte in der Schwangerschaft fortgeführt werden. Es können eine Bevorzugung nichthormoneller Kontrazeption sowie alternativ Gestagene empfohlen werden. Außerdem kann eine orale Kontrazeption mit einer Östrogen-Gestagen-Kombination bei Frauen mit inaktivem oder stabilem Lupus erythematodes ohne Antiphospholipidantikörper erwogen werden.

### S1-Leitlinie „Pyoderma gangraenosum“ [6]

Das Pyoderma gangraenosum ist eine seltene, teils sehr schwerwiegende und die Lebensqualität stark beeinträchtigende Erkrankung, die oftmals schwierig zu therapieren ist. Nicht selten müssen Therapeutika eingesetzt werden, die für das Pyoderma gangraenosum nicht zugelassen sind („off-label use“) und/oder für die keine kontrollierten Langzeitstudien vorliegen. Im September 2020 wurde erstmalig eine S1-Leitlinie zum Management des Pyoderma gangraenosum publiziert. Neben einer Darstellung der verfügbaren Daten zur Diagnostik und Therapie enthält diese auch einen übersichtlichen Managementalgorithmus.

### S1-Leitlinie „Analer Pruritus“ [7]

Pruritus ani (analer Juckreiz) ist ein in der ärztlichen Praxis und insbesondere im Kollektiv proktologischer Patient*innen häufiges und die Lebensqualität mitunter erheblich beeinträchtigendes Symptom. Die aktualisierte S1-Leitlinie „Analer Pruritus“ stellt eine lokalisationsspezifische Ergänzung zur S2k-Leitlinie „Diagnostik und Therapie des chronischen Pruritus“ [8] dar und enthält Empfehlungen zur Diagnostik und Therapie bei analem Pruritus. Die Klassifikation der Ätiologie ist grundlegend, um eine effiziente und nachhaltig wirksame Behandlung zu ermöglichen: In der Mehrzahl der Fälle tritt analer Juckreiz sekundär infolge einer anorektalen, dermatologischen oder sonstigen Pathologie oder Ursache auf; in diesen Fällen sind die kausale Behandlung und somit die Beseitigung bzw. Minimierung ursächlicher Faktoren essenziell. Aufgrund der Vielzahl an möglichen Ursachen empfiehlt sich ein abgestuftes, ggf. interdisziplinäres Vorgehen. In einigen Fällen lassen sich jedoch auch unter intensiven diagnostischen Bemühungen keine zugrunde liegenden Ursachen des analen Juckreizes identifizieren, es wird dann die Ausschlussdiagnose eines idiopathischen (primären) analen Juckreizes gestellt. Unabhängig von der ätiologischen Klassifikation sollten Allgemeinmaßnahmen zur Reduktion irritativer Reize im Bereich des Anus und Perianus empfohlen werden. Dies umfasst die Beratung zu einer schonenden Analhygiene, ggf. die Anpassung von Ernährungsgewohnheiten und die Pflege bzw. Protektion der perianalen Haut. Bei ausgeprägter oder unter den genannten Maßnahmen therapierefraktärer Symptomatik und insbesondere beim Vorliegen entzündlicher perianaler Hautbefunde kann eine kurzfristige oder intermittierende topische antientzündliche Therapie mit Kortikosteroiden oder Calcineurininhibitoren erforderlich sein. Weitere juckreizstillende Externa und Maßnahmen werden in der Leitlinie empfohlen.

### S1-Leitlinie „Diagnostik und Therapie des Analekzems“ [9]

Das Analekzem ist eines der häufigsten proktologischen Krankheitsbilder. Die 2019 aktualisierte S1-Leitlinie [9, 10] enthält Empfehlungen zum diagnostischen und therapeutischen Management. Neben dem Ausschluss relevanter Differenzialdiagnosen (z. B. Psoriasis intertriginosa, extramammärer Morbus Paget, AIN [anale intraepitheliale Neoplasie] und PAIN [perianale intraepitheliale Neoplasie]) ist die diagnostische Klassifikation des Analekzems entlang einer der 3 Hauptformen Grundlage einer sinnvollen und effektiven Behandlung: Das irritativ-toxische Analekzem entsteht infolge vermehrter perianaler Stuhlverunreinigung (z. B. bei Hämorrhoidalleiden oder chronischer Diarrhö), peranaler Sekretion (z. B. bei Fisteln oder rektaler sexuell übertragbarer Infektion) oder anderer chemischer/mechanischer Reize (z. B. intensive Analhygiene und häufiger Kontakt mit Detergenzien). Es ist Folge einer zugrunde liegenden anorektalen oder sonstigen Pathologie bzw. Ursache, ebenso wie das kontaktallergische Analekzem, das aufgrund einer Typ-IV-Reaktion auf externe Substanzen entsteht. Hiervon abzugrenzen ist das atopische Analekzem, das auf einer konstitutionell bedingten Störung der Hautbarriere beruht. Zu berücksichtigen sind darüber hinaus Überlappungsphänomene, bei denen es erst durch das Zusammenwirken unterschiedlicher Komponenten zu einem klinisch relevanten Befund kommt. Die Therapie des Analekzems umfasst beim irritativ-toxischen und beim kontaktallergischen Analekzem vordergründig die Behandlung bzw. Beseitigung ätiologischer Faktoren. Bei allen Formen des Analekzems kommen nichtmedikamentöse Therapieoptionen zum Einsatz. Diese dienen der Verminderung aggravierender Faktoren und bestehen aus einer Optimierung der Analhygiene und der Stuhlentleerungsgewohnheiten sowie Hautpflege und -protektion. Bei ausgeprägt entzündlichen Befunden sowie bei unzureichendem Erfolg der Beseitigung/Behandlung ätiologischer Faktoren und der Supportivmaßnahmen sollen zudem eine topische antientzündliche und/oder spezifische symptomatische Therapie durchgeführt werden. Dies umfasst den kurzzeitigen oder intermittierenden Einsatz topischer Glukokortikoide oder Calcineurininhibitoren. Im Falle von Superinfektionen kommen befundadaptiert zusätzlich Antiseptika, Antimykotika oder Antibiotika zum Einsatz.

### S2k-Leitlinie Neurodermitis – Aktualisierung zur Systemtherapie [11]

Aufgrund der fortlaufenden Entwicklungen im Bereich der Systemtherapie der Neurodermitis wurde das Kapitel zur Systemtherapie der S2k-Leitlinie basierend auf der wissenschaftlichen Datenlage und den konsentierten Empfehlungen aktualisiert.

Es wird empfohlen, die Indikation zur Systemtherapie der Neurodermitis ausreichend zu dokumentieren. Hierfür wird ein beispielhaftes Formular zur praktischen Verwendung zur Verfügung gestellt, das klinische Eignungskriterien für eine Systemtherapie berücksichtigt. Diese umfassen Kriterien zum objektiven Schweregrad sowie zum bisherigen (fehlenden) Therapieansprechen. Ebenso wird die subjektive Belastung berücksichtigt, und dabei werden therapieresistente Ekzeme an sichtbaren Körperstellen wie im Gesicht oder an den Händen als schwere Form der Neurodermitis eingestuft.

Im Hinblick auf das aktuelle Therapiespektrum wurde Dupilumab in die Leitlinie neu aufgenommen. Ein starker Konsens der Mandatsträger*innen lag dafür vor, dass der Einsatz von Dupilumab zur Therapie der chronischen, moderaten bis schweren Neurodermitis von Jugendlichen ab 12 Jahren und bei Erwachsenen, die mit topischen Medikamenten alleine nicht ausreichend behandelt werden können, empfohlen werden kann. Damit steht nun erstmals auch ein Systemtherapeutikum mit Zulassung im Kindesalter zur Verfügung.

Die Aufnahme dieser neuen, gleichberechtigten First-line-Therapieoption hatte zudem eine Neubewertung der sonstigen Systemtherapien bei Neurodermitis zur Folge, und das Schema zur Stufentherapie bei Neurodermitis wurde entsprechend angepasst. Die Empfehlungsstärke von Ciclosporin wurde dabei im Vergleich zur Vorversion dieses Leitlinienkapitels auf die schwächste Positivempfehlung herabgestuft („kann erwogen werden“). Darüber hinaus sind notwendige Voruntersuchungen vor Therapieeinleitung gezielter und auch Empfehlungen für den Off-label-Einsatz von Ciclosporin unter einem Alter von 16 Jahren formuliert worden.

Die – nach wie vor schwächste – Positivempfehlung für den Einsatz von Mycophenolat-Mofetil bezieht sich nunmehr auf Einzelfälle, und der Aspekt der Teratogenität mit Konkretisierung der Kontraindikationen ist Teil der entsprechenden Empfehlungsbox.

Während die Empfehlungsstärke (schwächste Positivempfehlung) hinsichtlich einer Systemtherapie mit Glukokortikosteroiden unverändert geblieben ist, sind in der Leitlinie nun die diesbezügliche Therapiedauer und Dosierung konkret definiert (Kurzzeittherapie mit oralen Glukokortikosteroiden, d. h. wenige Wochen, Dosis ≤ 0,5 mg/kgKG [Körpergewicht] Prednisolonäquivalent). Ein starker Konsens liegt dafür vor, dass wegen der unerwünschten Arzneimittelwirkungen eine längerfristige Therapie der Neurodermitis mit systemischen Glukokortikosteroiden weiterhin nicht empfohlen wird.

## Ausgewählte deutsche Leitlinien unter Federführung anderer Fachgesellschaften

### S3-Leitlinie „Diagnostik, Therapie und Nachsorge von Analkanal- und Analrandkarzinomen“ [12]

Das Analkarzinom zählt zu den seltenen malignen Tumoren, jedoch ist die Inzidenz in den letzten Jahren kontinuierlich angestiegen. Die S3-Leitlinie „Diagnostik, Therapie und Nachsorge von Analkanal- und Analrandkarzinomen“ definiert erstmals in Deutschland Empfehlungen für das Management des Analkarzinoms auf Grundlage einer evidenz- und konsensbasierten Leitlinienmethodik. Eine für proktologisch tätige Dermatolog*innen relevante Neuerung besteht in der Empfehlung, zytologische Screeninguntersuchungen auf Analkarzinome nicht nur Menschen, die mit HIV („human immunodeficiency virus“) leben, anzubieten, sondern auch anderen Personengruppen mit erhöhtem Risiko (z. B. Frauen nach genitalen Dysplasien/Karzinomen; Organtransplantierten und anderen erheblich Immunkompromittierten; Männern mit Angabe von rezeptivem Analverkehr mit häufig wechselnden Partnern). Die initiale Diagnostik umfasst neben der Dokumentation von Lage und Ausdehnung des Primärtumors die histopathologische Probengewinnung und die Durchführung klinischer, instrumenteller und bildgebender Untersuchungen. Bei Verdacht auf Analrandkarzinom von bis zu 2 cm Durchmesser ohne Infiltration des Sphinkterapparats oder benachbarter Organe sollte bereits zum Zeitpunkt der Diagnosesicherung eine therapeutische R0-Exzision mit Sicherheitsabstand (0,5 cm) angestrebt werden. Die Behandlung von Analkarzinomen erfolgt stadienadaptiert und entsprechend der Lokalisation des Primärtumors: Während Analrandkarzinome im Stadium I mit Sicherheitsabstand lokal exzidiert werden sollen, ist die kombinierte Radiochemotherapie mit Mitomycin und 5‑FU (5-Fluoruracil) nach wie vor Therapiestandard bei Analrandkarzinomen im Stadium II bis III und Analkanalkarzinomen im Stadium I bis III. Im Rahmen der Radiochemotherapie kann Mitomycin durch Cisplatin und 5‑FU durch Capecitabin ersetzt werden. Die Response-Evaluation soll zu verschiedenen Zeitpunkten erfolgen; dabei soll bei residuellem lokalem Tumorbefund ohne klinischen Anhalt für Progress die weiterführende Diagnostik frühestens 26 Wochen nach Beginn der Radiochemotherapie erfolgen. Nach Behandlung in kurativer Intention ist eine 5‑jährige protokollgesteuerte Nachsorge anzubieten.

### S3-Leitlinie „Impfprävention HPV-assoziierter Neoplasien“ [13]

Die HPV(humane Papillomviren)-Impfung stellt einen effektiven und sicheren Schutz vor benignen und malignen HPV-assoziierten Läsionen dar, insbesondere vor anogenitalen Warzen, intraepithelialen Neoplasien und Karzinomen, aber auch vor HPV-assoziierten Oropharynxkarzinomen. In der Leitlinie wird empfohlen, dass alle Kinder unabhängig von ihrem Geschlecht im Alter von 9 bis 17 Jahren, möglichst frühzeitig, gegen HPV geimpft werden sollen. Dies ist kongruent mit den Empfehlungen der Ständigen Impfkommission (STIKO) am Robert Koch-Institut (RKI). Ergänzend hierzu enthält die Leitlinie praktische Empfehlungen zu Auffrischimpfungen sowie zur Austauschbarkeit der Impfstoffe und der Möglichkeit der zusätzlichen Impfung nach bereits erfolgter Impfung.

In der Praxis stellt sich häufig die Frage nach dem Einsatz der Impfung bei Personen über dem 18. Lebensjahr. Basierend auf den verfügbaren Studiendaten, empfiehlt die Leitliniengruppe, dass HPV-impfnaive Erwachsene im Alter von 18 bis 26 Jahren unabhängig von ihrem Geschlecht gegen HPV geimpft werden sollten. Personen im Alter von 27 Jahren oder älter sollte die Impfung jedoch nicht empfohlen werden. Darüber hinaus sind in der vorliegenden Leitlinie Empfehlungen zur Impfung bei bestimmten Risikopopulationen enthalten, z. B. bei HIV-positiven Patienten, anderweitig immunkompromittierten Patienten sowie Personen, die planen, die HIV-Präexpositionsprophylaxe (PrEP) einzunehmen.

Eine HPV-Impfung mit dem Ziel eines therapeutischen Nutzens im Rahmen der Behandlung bestehender HPV-assoziierter Läsionen sollte laut der Leitliniengruppe nicht durchgeführt werden. Bei HPV-impfnaiven Frauen mit zervikalen intraepithelialen Neoplasien (CIN) kann allerdings eine HPV-Impfung vor oder nach der Behandlung der CIN mit dem Ziel einer Reduktion der Wiedererkrankungsrate erwogen werden.

Unabhängig vom Alter und Geschlecht soll eine HPV-Testung zur Entscheidungsfindung vor einer Impfung nicht erfolgen. Die HPV-Testung und -Typisierung ist von sehr limitierter Aussagekraft hinsichtlich der Frage nach dem Nutzen der HPV-Impfung nach der Aufnahme sexueller Kontakte. Früherkennungsuntersuchungen (Zervixkarzinomscreening) einschließlich der ab dem Alter von 35 Jahren angebotenen kombinierten HPV-Testung sollten entsprechend den Empfehlungen der S3-Leitlinie Prävention des Zervixkarzinoms [14] und den Vorgaben der Richtlinie des Gemeinsamen Bundesausschusses für organisierte Krebsfrüherkennungsprogramme unabhängig vom HPV-Impfstatus angeboten werden.

Erstmalig enthält die Leitlinie auch Empfehlungen für organisatorische Maßnahmen zur Verbesserung der bislang unzureichenden Implementierung der HPV-Impfung in Deutschland. Diese Empfehlungen wenden sich primär an Gremien, die über solche organisatorischen Maßnahmen und deren Umsetzung entscheiden können, und nicht an individuelle Anbieterinnen und Anbieter von Gesundheitsdienstleistungen.

### S2k-Leitlinie „Diagnostik und Therapie der Syphilis“ [15]

Ende April 2020 wurde die Aktualisierung der S2k-Leitlinie „Diagnostik und Therapie der Syphilis“ publiziert. Während im Vergleich zur Vorversion die Empfehlungen zur Behandlung der Früh- und Spätsyphilis im Wesentlichen unverändert sind, enthält die Aktualisierung der Leitlinie neue Empfehlungen zur Diagnostik. So wird bei Verdacht auf einen frühen Primäraffekt eine schwache Empfehlung zur ergänzenden Durchführung eines direkten Erregernachweises mittels PCR(Polymerasekettenreaktion)-Diagnostik (Abstrich aus dem Ulkus) ausgesprochen. Dieser bietet als Ergänzung zur serologischen Syphilisdiagnostik den Vorteil eines kürzeren diagnostischen Fensters und ermöglicht so mitunter eine raschere Diagnosestellung und Therapieeinleitung. Dabei ist zu beachten, dass ein negativer PCR-Abstrich eine aktive Syphilis nicht ausschließt und isoliert positive PCR-Befunde bei negativer Serologie durch serologische Verlaufskontrollen hinsichtlich ihrer Plausibilität zu überprüfen sind. Serologische Kontrollen des Therapieerfolges sollen weiterhin nach 3, 6 und 12 Monaten erfolgen – neu ist der Zusatz, dass die Behandlung bei Abfall des Cardiolipin (VDRL [Venereal Disease Research Laboratory] oder RPR [Rapid-Plasma-Reagin])-Antikörpertiters um mindestens 2 Stufen als erfolgreich betrachtet und auf die weiteren Titerkontrollen verzichtet werden kann.

Des Weiteren wurde im Rahmen der Konsensuskonferenz die präventive Doxycyclin-Einnahme zur Prophylaxe von Syphilisinfektionen bei Personen mit hohem Risiko diskutiert. Patienten fragen zunehmend danach, und es liegen randomisierte Studien vor. Aufgrund der bislang unvollständigen Datenlage im Hinblick auf die Auswirkungen einer solchen Strategie auf Resistenzen bakterieller Mikroorganismen wird der Einsatz einer Doxycyclin-Prophylaxe zur Prävention von Syphilisinfektionen äußerst kritisch bewertet. Die Leitlinienkommission hat daher beschlossen, zum aktuellen Zeitpunkt keine entsprechende Empfehlung auszusprechen. Davon unberührt bleibt die schwache Empfehlung, Personen nach relevantem Erregerkontakt eine Postexpositionsprophylaxe analog zur Behandlung der Frühsyphilis anzubieten.

## Ausgewählte europäische Leitlinien

### European S1 guidelines on the management of lichen planus [16]

Mit dem Lichen planus hat sich die Leitliniengruppe des European Dermatologie Forums um Dimitrios Ioannides eine Erkrankung zum Inhalt genommen, die bisher noch wenig im Fokus neuer Therapieentwicklungen stand und oftmals schwer zu behandeln ist. Die Erstellung der Leitlinie entstand als europäischer Expertenkonsens und listet eine Vielzahl möglicher Therapieoptionen auf. Fast alle Therapieoptionen sind nicht für die Behandlung des Lichen planus zugelassen.

Für die kutane Manifestation des Lichen planus werden topische Steroide, intraläsionales Triamcinolon, systemische Steroide, Acitretin und auch systemisch angewendetes Cyclosporin als Erstlinienbehandlung empfohlen. Als Zweitlinientherapie finden UV-Therapien, ggf. in Kombination mit Acitretin, topische Calcineurininhibitoren sowie Sulfasalazin Erwähnung.

Bei einer Manifestation des Lichen ruber an der Schleimhaut unterscheiden sich die Erstlinientherapieempfehlungen nicht wesentlich von denen für die Behandlung einer kutanen Manifestation.

Für die Behandlung eines Lichen planopilaris finden in der Erstlinienbehandlung neben den Steroiden Cyclosporin, Hydroxychloroquin, Methotrexat und topische Calcineurininhibitoren Erwähnung.

Die Behandlung des Lichen ruber der Nägel stellt in der Regel eine besondere Herausforderung dar. Bei 50 % der Patienten ist laut Leitlinie keinerlei Ansprechen auf jedwede Therapie zu erwarten. In der Leitlinie wird als Erstlinientherapie die in Deutschland eher unübliche intramuskuläre Gabe von Triamcinolon empfohlen, alternativ die sehr schmerzhafte intraläsionale Injektion von Triamcinolon. Angenehmer für den Patienten ist die ebenfalls empfohlene orale Einnahme von Prednisolon oder die okklusive topische Anwendung. Als weitere zu erwägende systemische Therapieoptionen werden Alitretinoin, Chloroquin, Cyclosporin, Acitretin, Biotin, Etanercept und topisch Tacrolimus-Salbe genannt.

Wichtig ist vor der Therapie das Prüfen möglicher medikamentöser Auslöser, z. B. ACE(Angiotensin-Converting-Enzym)-Hemmer, Betablocker, Nifedipin, HCT (Hydrochlorothiazid), Antikonvulsiva, Tuberkulostatika, Malariamedikamente.

## Fazit für die Praxis

- Aktuell steht eine Vielzahl an Leitlinien als Hilfestellung zu verschiedensten Krankheitsbildern zur Verfügung.
- Aufzufinden sind die deutschen Leitlinien unter www.awmf.org bzw. die europäische Leitlinie auf den Homepages der jeweils herausgebenden Vereinigungen (z. B. EDF/European Centre for Guidelines Development: www.edf.one/home/Guidelines/EDF-EuroGuiDerm.html).
- Jede Leitlinienempfehlung muss unter Berücksichtigung der individuellen Situation des Patienten geprüft, individualisiert und ggf. an den Patienten angepasst umgesetzt werden.
